# Machine learning framework for precise localization of bleached corals using bag-of-hybrid visual feature classification

**DOI:** 10.1038/s41598-023-46971-7

**Published:** 2023-11-09

**Authors:** Fawad, Iftikhar Ahmad, Arif Ullah, Wooyeol Choi

**Affiliations:** Department of Computer Engineering, Chosun University, Gwangju, 61452 Republic of Korea

**Keywords:** Ocean sciences, Computer science

## Abstract

Corals are sessile invertebrates living underwater in colorful structures known as reefs. Unfortunately, coral’s temperature sensitivity is causing color bleaching, which hosts organisms that are crucial and consequently affect marine pharmacognosy. To address this problem, many researchers are developing cures and treatment procedures to restore bleached corals. However, before the cure, the researchers need to precisely localize the bleached corals in the Great Barrier Reef. The researchers have developed various visual classification frameworks to localize bleached corals. However, the performance of those techniques degrades with variations in illumination, orientation, scale, and view angle. In this paper, we develop highly noise-robust and invariant robust localization using bag-of-hybrid visual features (RL-BoHVF) for bleached corals by employing the AlexNet DNN and ColorTexture handcrafted by raw features. It is observed that the overall dimension is reduced by using the bag-of-feature method while achieving a classification accuracy of 96.20% on the balanced dataset collected from the Great Barrier Reef of Australia. Furthermore, the localization performance of the proposed model was evaluated on 342 images, which include both train and test segments. The model achieved superior performance compared to other standalone and hybrid DNN and handcrafted models reported in the literature.

## Introduction

Coral reefs are considered the world’s most diverse marine ecosystem. The coral reefs host an estimated 25% of overall marine life, in which more than 4000 species of fishes exist^1^. The presence of parasitic algae (zooxanthellae) in the coral creates a colorful calcium carbonate structure underwater, commonly known as reefs. Coral reef bleaches when algae evacuate from coral’s tissue with an increase in the water temperature^2^. Coral reef bleaching is associated with diverse economic and environmental issues. Global warming is the leading cause of bleaching because the sea surface temperature (SST) increases unusually during the summer season^3^. In 2016, 29 to 50% of the corals were killed by bleaching on the Great Barrier Reef in Australia^4^. Furthermore, the CO$_2$ level in the world’s oceans is increasing day by day because of bleaching, which makes the environment more acidic, and hence creates difficulties for other corals and marine organisms in building their skeleton. Reef hosts various marine species with a lot of pharmaceutical compounds that are helpful in the treatment of many world’s dangerous diseases^5^.

The marine ecosystem needs regular monitoring and survey to reduce the effects of climate change on it. The presence of artifacts and environmental noises in the underwater imagery makes it difficult for the computer vision algorithm to distinguish between the background and the foreground desired object. Therefore, underwater image enhancement methods have been developed^6^. The integrated color model (ICM) and the unsupervised color correction method (UCM)^7^ enhance contrast by converting images into the HSI model and then stretching the saturation and intensity components of the image. Artificial intelligence (AI) researchers aim to develop a robust and computationally efficient algorithm to locate bleached coral reefs. However, their localization models suffer performance degradation due to variations in illumination, scale, orientation, viewpoint, occlusions, and background clutters. The variations in the object’s scale, viewpoint, and illumination are due to the camera’s depth, mount position, and variation in the light sources in the surveillance environment, respectively. The researchers aim to develop handcrafted and deep-learning feature extraction techniques that are robust to the geometric and appearance variations that occur in marine ecosystem images. The geometric features highly depend on the local distribution of contours and edges that make the shape of objects within the image, whereas the appearance-based feature comprises the texture and color details of the object in the image. Both geometric and appearance features are sensitive to variations in illumination, scale, orientation, viewpoint, occlusions, and background clutter^8^.

Nowadays, deep neural network (DNN) models replaced the traditionally handcrafted feature extractors in most classification tasks. The DNNs such as ResNet, DenseNet, VGGNet, and Inceptions models are domain-independent and are trained on a large number of datasets, thus providing unrivaled success in diverse tasks. The DNN suffers over-fitting due to fewer bleached samples in the existing databases, thus affecting the robustness and distinctiveness of the features.

On the other hand, the handcrafted feature’s robustness and distinctiveness have no concern with the training data’s strength. Regardless of noise robustness, the handcrafted feature’s invariance is still affected by variations in depth, underwater illuminations, and water turbidity. The work aims to develop an invariant feature extraction model that remains robust to geometric and photometric changes within the coral images. The proposed framework extracts raw features with a hybrid handcrafted and DNN approach followed by the BoF to reduce and bring further invariance that improves classification accuracy. To enhance photometric invariance, the proposed model relies on the image’s local features instead of global features. Furthermore, the Bag-of-features adoption in the proposed architecture reduces the dimension of the raw hybrid feature vector, thus reducing the storage requirement and complexity. The best-performing classifier, kernel combination, optimum patch, and cluster size have been identified through extensive experimentation.

## Related work

Computer vision has played a vital role in the automatic annotation and classification of marine images. Convolutional neural networks (CNNs) and many handcrafted models have been developed for the analysis and classification of underwater marine images. In the literature, the handcrafted features for corals classification have been focused on the color and texture of the coral images. In Ref.^9^, the authors developed a color descriptor over multiple scales moorea labeled corals (MLC) images, which yielded a classification accuracy of 83.1% and outperformed the other color texture descriptors in this domain. In Ref.^10^, an improved local directional pattern (ILDP) is developed that captures diagonal pattern features based on the variation of local derivative. ILDP constituting of support vector machine (SVM), K-nearest neighbor (KNN), and CNN classifiers has achieved superior performance compared to LBP, LDP, LTRP, and RLTP models when validated on EILAT, RSMAS, and MLC-2012 datasets. In Ref.^11^, two novel mapping methods Riu1 and Riu2 of LBP, CLBP, and LTP models have been validated on EILAT-2, RSMAS, and MLC-2008 corals datasets for the extraction of discriminative features from the texture.

In the recent past, CNN models have grasped the attraction of underwater imagery study and marine object recognition and detection. Hence, replacing the traditional handcrafted feature models. Researchers have investigated SVM, MLP, and CNN to categorize the unlabeled coral mosaics of the Abrolhos Islands, Australia. The three CNNs models ResNet, Inception-v3, and DenseNet are studied in^12^ for the classification of RSMAS and EILAT coral datasets close-up images, and the imbalance problem in the loss function was also highlighted. Among them, the ResNet model performed better for the stated datasets. Similarly, based on the work in^12^, for the smaller dataset, simple model such as ResNet-50 outperform the complex models such as DenseNet-161 and DenseNet-121. The authors in^13^ classified 3 plankton and 2 coral datasets using DNN models such as AlexNet, VGGNet, Inception-v3, GoogleNet, ResNet, and DenseNet. These models were fine-tuned and combined with an ensemble which resulted in improved performance compared to the individual model. In Ref.^14^, a fluorescence imaging system (FluorIS) is used to capture fluorescence images with a wider band and field of view. The annotation of fluorescence images is done with the CNN model, and a 22% error rate reduction is achieved compared to simple Florence image classification. In Ref.^15^, five patches-based CNN and four fully connected CNN (FCNNs) approaches are used for the segmentation and classification of the coral reef images. Among the five CNN models, the ResNet-152 performed best, whereas the DeeplabV2 outperformed the other three FCNN architectures for the corals’ dataset. Overall, the patch-based CNN’s classification accuracy is higher than FCNN models. In Ref.^16^, these conventional patch-based CNN and FCNN models are extended by adding extra layers to incorporate multi-view stereoscopic input image data and improve the semantic segmentation and classification accuracy. However, the nViewNet-8 and TwinNet architectures developed in^16^ outperform the proposed patch-based CNNs and FCNN models.

Some research works integrate handcrafted feature extractors with CNNs to form hybrid feature extraction models. The existing hybrid frameworks are capable of enhancing the photometric invariance of their model by concatenating the colors, shapes, and texture features of the input image. However, the huge dimension of the final feature vector affects the computational complexity. CNN-based features extracted from the same patches were concatenated with handcrafted features using the MLC benchmark dataset of corals. In Ref.^17^, close-up images of coral reef components were classified into live corals, dead corals, and sand using a neural network that utilizes color and texture-based joint features as an input. The hue saturation (HS) and RGB color features are combined to rotational invariant local binary patterns with uniformity less than 2 (LBPriu2) textures for input image representation. However, such integration increases the feature dimension at the cost of memory allocation. To deal with these issues, this paper focuses on proposing a robust localization framework for bleached corals using BoHVF.

### Contribution and paper organisation

The noise-robustness and photometric-invariance of the existing DNN methods are limited due to the availability of fewer amount of training samples. To deal with training samples limitation, in this paper, the handcrafted method is considered a hybrid part of the existing DNN model. The main contribution of this paper is listed as follows.

- Extensive analysis: In this work, we proposed a hybrid framework for the localization of corals that utilizes ColorTexture^18^ along with ALexNet^19^ to robustly extract the features while classification is done using SVM with a Quadratic kernel. Our experiments show that our proposed hybrid framework provides higher performance with handcrafted color texture concatenated with the AlexNet DNN model.
- Bag-of-Features strategy: The dimension of the raw feature set is reduced up to 16 times by applying the bag-of-features method over the raw hybrid feature set.
- Patch description: The novel patch-based representation is adopted to describe the local features instead of global features. Furthermore, the patch dimension is decided through extensive experiments.

The rest of the paper is organized as follows. The proposed Methodology including the feature representation and classification is discussed in Methodology. The dataset and experimental results are discussed in Results and Discussion, and finally, the paper’s conclusive remarks are presented in Conclusion.

**Figure 1:**
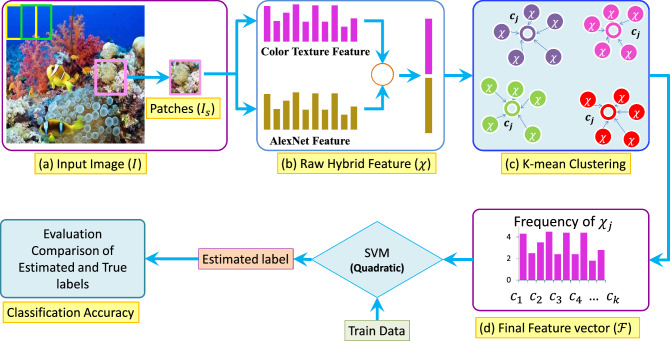
Proposed bag-of-hybrid visual features framework.

## Methodology

In this section, we present the detailed methodology of the proposed approach. We present the dimension reduction of the raw feature set followed by the final feature representation and classification.

The proposed methodology consists of two prime stages including the feature reduction through the bag-of-feature (BoF) approach and its classification given in Fig. 1. Each input image *I* is divided into small patches $I_s$ of dimension $M\times M$, and the raw features are extracted from the consecutive patches with a 50% overlapped between them. The patches $I_s(i, j)$ can be mathematically expressed as

1 $$\begin{aligned} I_s(i, j)&= \Bigg \{{\textbf{I}}\left( i\frac{M}{2}+\omega ,j\frac{M}{2}+\omega \right) \Bigg \}, \nonumber \\&\quad \forall i \in \Big \{0,... , \frac{2}{M} \left( L_r-\omega \right) \Big \}, \forall i \in \Big \{0,... , \frac{2}{M} \left( L_c-\omega \right) \Big \}, \end{aligned}$$

where *M* denotes the dimension of the patch $\vec {I_s(i, j)}$, $\omega$ is the index value ranges between *M* and 1, $L_r$ is the total number of rows, and $L_c$ is the total number of columns in image ${\textbf{I}}$.

To extract the vocabulary data, a raw feature set is obtained from all patches of the coral images belonging to the training set. The same set of handcrafted and deep feature extraction models is also used in the test BoF feature extraction process. Once the handcrafted raw feature set is extracted, the DNN model is further reduced through the BoF model. The texture consists of a uniform spatial distribution of textons. The local neighbor distribution of pixel intensities in the RGB channels robustly represents the repetition of textons. ColorTexture processes the image through the quantization of its color into RGB channels and collecting local histograms from its patches. These histograms are then concatenated to represent the image content in a distinctive format. The raw hybrid features vector extracted from the patch ${\textbf{I}}_s$ of the input image is given as

2 $$\begin{aligned} {\vec {\varvec{\chi }} = \Big [\vec {{\mathcal {A}}({I_s})} ~\vec {{\mathbb {C}}(I_s)} \Big ]^T, } \end{aligned}$$

where $\vec {{\mathcal {A}}(I_s)}$, and $\vec {{\mathbb {C}}(I_s)}$ denote the feature vectors generated by the AlexNet DNN model, and the color-texture descriptor, respectively, while $[\cdot ]^T$ denotes the transpose of features vector.

To extract the low dimensional feature vector, the vocabulary set is employed in the image representation stage of the proposed BoF model. The BoF relies on the visual vocabulary data, which is obtained from the raw feature set by the clustering approach. Hence, the k-mean clustering is used to determine the centroid of the raw feature set. The vocabulary set is determined through k-mean clustering of the raw features representing the patches of the overall training set. The ${\mathcal {V}}$ number of cluster centroids, which are the visual vocabulary sets in the BoF method based on the clustering of the objective function, ${\mathcal {L}}$, and is written as

3 $$\begin{aligned} {{\mathcal {L}} = \sum _{i=1}^{n} \sum _{j=1}^{m} \psi _{ij}||\vec {\chi _i }- c_j ||^2,} \end{aligned}$$

where $c_j$ represents the centroid of the *jth* cluster, $\vec {\chi _i}$ denote the *ith* raw feature vector in the training data set, $\psi _{ij}$ is the indicator variable that assigns *ith* feature vector to the *jth* cluster based on the minimum Euclidean distance, *n* is the total number of raw features in the training data set, and *m* denotes the size of the cluster.

### Final feature representation

The raw hybrid features consisting of handcrafted and deep features concatenated have a high dimension that is reduced through the BoF method. The proposed RL-BoHVF extracts final features through the visual vocabulary set $c_j$ obtained through the clustering process. A ${\mathcal {V}}$ number of the raw feature clusters is created through k-mean clustering. The cluster centers are considered vocabulary sets and determine the dimension of the final feature vector. The higher value of ${\mathcal {V}}$ leads to a high dimensional final feature vector and vice versa. The final feature vector is the frequency distribution of the cluster centers in the raw feature set of the input image’s patches. The final feature extraction process consists of two stages. Initially, the raw hybrid features are extracted from the input image patches. Then the frequency of each cluster center is determined in the raw feature set. The histogram of vocabulary vectors represents the final feature vector. In this case, we have *m* clusters from the bleached data, while n features from the unbleached data, which makes a total of $m+n$ feature values in the final feature vector. The final reduced feature vector, $\vec {{\mathcal {F}}(j)}$, can be written as

4 $$\begin{aligned} \vec {{\mathcal {F}}(j)}&= {\left\{ \begin{array}{ll}\sum \limits _{i=1}^{N}\tau (\chi _i,c_j),~\hspace{0.2in}\text {if}~ || \chi _i-c_j ||^2 \le \eta _i, \\ 0,~\hspace{0.83in}\text {Otherwise},\end{array}\right. } \end{aligned}$$

5 $$\begin{aligned} \eta _i&= \frac{\sum _{j=1}^{m} || \chi _i-c_j ||^2}{m} \end{aligned}$$

where $\eta _i$ in Eq. (5) is the minimum threshold value to decide whether the feature vector matches the centroid, and $\tau$ is the unit step function whose value remains 1 when $\chi _i$ and $c_j$ match each other and 0, otherwise. The reduced feature set of all test images is classified with the SVM classifier into binary classes. The details of the classification approach are further presented in the next sub-sections.

### Classification

Based on the image-based classification problem, the images that contain bleached corals are considered positive, while the images that do not contain bleached corals are considered negative images. The SVM classifies the final feature vector extracted from the test set into positive or negative classes. Various kernels of the SVM classifier including the Linear, Quadratic, Cubic, and Gaussian are used to categorize the visual features into bleached and unbleached classes. A set of train data consisting of a combination of extracted features along with class labels is used to classify the test features into their respective category. The set of actual and estimated labels is used to calculate the classification accuracy of the overall model.

**Figure 2:**
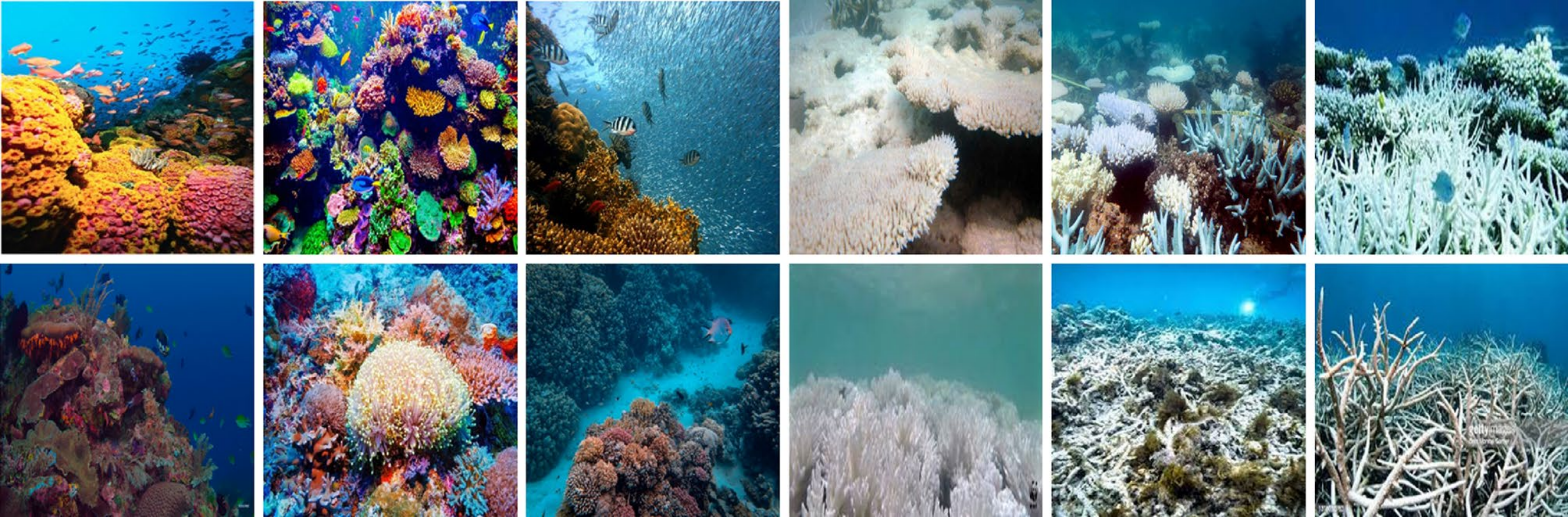
Sample images of the dataset: the left three columns are normal corals while the right three columns are bleached corals.

**Figure 3:**
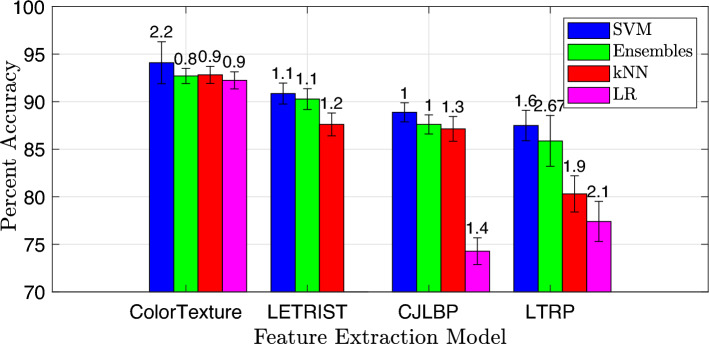
Classification performance of the handcrafted model on various classifiers. The corresponding standard deviation values are mentioned on each percent accuracy bar.

## Results and discussion

Using the proposed framework, we performed extensive simulations. The detail of the dataset and parameters followed by the simulation results and discussion are presented next.

### Dataset description

The dataset consists of ${\mathcal {T}}$ number of coral images collected from the Great Barrier Reef in Australia^4^, which comprises bleached and unbleached coral images. Out of the total images, ${\mathcal {B}}$ number of images are bleached corals while ${\mathcal {U}} = {\mathcal {T}}-{\mathcal {B}}$ number of images are unbleached corals. The dataset contains challenging attributes such as low resolution with variations in illumination, view angle, scale, and orientation with a set of occluded and background clutter effects. Some bleached corals closely resemble stones and woods with a high resemblance to outliers. Moreover, due to low-resolution dust particles and other obstacles, some corals closely resemble small colorful marine animals. The variations in illumination and scale are due to depth variations in the ocean. Similarly, the angular variations are due to the presence of rock surfaces in water. The undersea rocks, bushes, and marine animals caused occlusion in the captured images. Furthermore, the image resolution depends on the quality of the camera, water transparency, etc. A sample of bleached and unbleached images from the dataset is shown in Fig. 2.

### Simulation parameters

**Figure 4:**
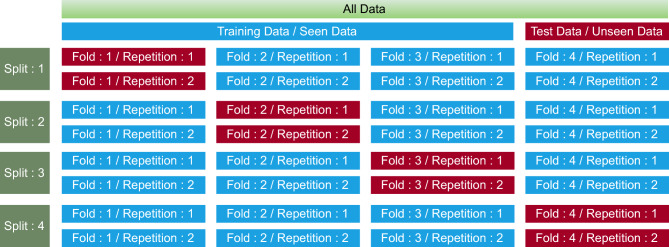
K-fold cross validation method.

In the simulation, the dataset has ${\mathcal {T}} = 342$ coral images which has ${\mathcal {B}} = 184$ and ${\mathcal {U}} = 158$ number of bleached and unbleached coral images, respectively. During the training of the proposed framework, *K*-fold validation with $K = 4$ is used for cross-validation. Moreover, 75% of the input data with bleached and unbleached images is used for training while the rest of 25% is used for testing of both classes, respectively. Figure 4 shows that within each iteration, the data is split into training and test segments, where 75% of the data is used for training and 25% of unseen data is used for testing. Each iteration, including the same data split, is repeated two times. In each iteration, a different unseen fold is selected for testing, while the remaining three folds are used to train the model. To split each image into an equal number of patches, all the images in the dataset are resized to a uniform dimension of size $512 \times 512$. Furthermore, for feature extraction, the images are split into $M \times M$ overlapped patches with a maximum of 50% of overlap between the two consecutive features. The same approach has been used for all *K*-fold validation procedures, and a total of two repetitions were performed during each fold and a bag contains $2 {\mathcal {V}}$ number of vocabulary where ${\mathcal {V}}$ denotes scenario dependent cluster size. The performance of the proposed RL-BoHVF in terms of overall classification accuracy concerning different parameters is presented in the following sections.

### Results

The dataset contains images belonging to two classes: bleached coral and unbleached corals. These images exhibit variations in view angle, scale, depth, and illumination setup. Additionally, the presence of rocks and marine animals in the dataset introduces noise, such as occlusions and background clutter, as depicted in Fig. 2.

**Figure 5:**
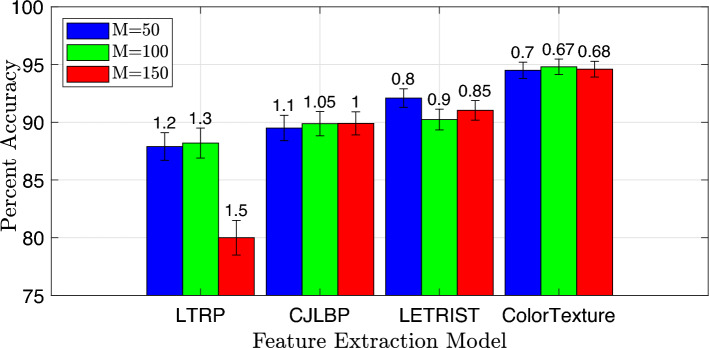
Classification accuracy (%) comparison of feature extraction models with respect to various segment size (*M*) for ${\mathcal {V}} = 100$. The corresponding standard deviation value for each method is mentioned on each bar.

To generate raw features from the input image data, we experimented with multiple handcrafted and DNN models. The list of handcrafted methods for raw features extraction include locally encoded transform feature histogram (LETRIST)^20^, completed joint-scale local binary pattern (CJLBP)^21^, and local tetra pattern (LTP)^22^ while the DNN models that are evaluated for raw feature extraction include Inception-v3^23^, ResNet-50^24^, AlexNet^19^ and GoogleNet^25^. During experiments on the aforementioned handcrafted methods, the one with the best performance was tested in combination with the listed DNN models. We observed that the optimal performance can be achieved by considering a combination of handcrafted ColorTexture and the DNN AlexNet model. The ColorTexture handcrafted model employed in the proposed model consists of texture along with color quantization to bring robustness and invariance to the final feature vector. To select the best-performing classifier, classification kernel for the best classifier, patch size, and cluster size, we first evaluate the performance of the handcrafted feature extraction approaches in Fig. 3, Tables 1, 2, and Fig. 5, respectively.

The results are tested for various descriptors, different classifiers with varieties of kernels, and a range of ${\mathcal {V}}$ and *M* values. In Fig. 3, the performance of different classifiers such as SVM, Ensemble, KNN, and logistic regression (LR) for different features extraction models including ColorTexture, LETRIST, CJLBP, and LTRP is compared in terms of percent accuracy. The best-performing classification model identified in Fig. 3 is further used with different kernels to detect the highly precise kernel type, as given in Table 1. The robust classifier and the best-performing kernel type identified in the aforementioned evaluation are further tested for various cluster sizes ranging from ${\mathcal {V}} = 10$ to ${\mathcal {V}} = 100$, to detect the most precise cluster size as given in Table 2. Figure 5 presents the classification performance when tested on various patch sizes to detect the optimum patch value.

For the input image with $M = 100$ and ${\mathcal {V}} = 100$, for a given feature extraction model and dataset, the SVM classifier outperformed the rest of the classifiers. Furthermore, for the given set of classifiers, the ColorTexture descriptor shows high accuracies compared to LETRIST, CJLBP, and LTRP. Figure 3 illustrates the classification accuracy achieved by the SVM, Ensemble, KNN, and LR methods using the ColorTexture model. Specifically, the SVM achieves an accuracy of 93.80%, the Ensemble achieves 92.50%, the KNN achieves 92.60%, and the LR achieves 92.20%. These accuracies are accompanied by corresponding standard deviations (STD) of 2.2, 0.8, 0.9, and 0.9 for the SVM, Ensemble, KNN, and LR methods, respectively. The results given in Fig. 3 show that the SVM classifier outperformed Ensemble, KNN, and LR with an accuracy difference of 1.30%, 1.20%, and 1.60%, respectively. The SVM with the ColorTexture provides the highest accuracy compared to the other classifiers and feature extraction models, i.e. LETRIST, CJLBP, and LTRP with an accuracy difference of 3%, 5%, and 6.49%, respectively.

**Table 1:** Classification accuracy (standard deviation) of the handcrafted model with various combinations of SVM kernels.

SVM kernel	ColorTexture	LETRIST	CJLBP	LTRP
	Ref.^18^	Ref.^20^	Ref.^21^	Ref.^22^
Linear	92.60 (2.4)	90.80 (3.2)	87.60 (3.3)	81.70 (2.8)
Quadratic	93.80 (1.3)	90.80 (1.6)	88.80 (1.6)	87.31 (2.5)
Cubic	91.00 (2.7)	70.30 (4.1)	62.20 (6.2)	60.40 (5.7)
Fine Gaussian	85.50 (3.8)	84.90 (2.0)	82.30 (3.0)	77.50 (6.2)
Coarse Gaussian	92.80 (1.7)	79.30 (3.9)	85.50 (2.5)	74.70 (6.8)
Medium Gaussian	92.40 (1.9)	75.50 (3.2)	79.10 (3.8)	73.70 (6.9)

**Table 2:** Classification accuracy (standard deviation) of descriptors with respect to different cluster sizes.

Descriptor name	${\mathcal {V}} =10$	${\mathcal {V}} =25$	${\mathcal {V}} =50$	${\mathcal {V}} =100$
ColorTexture^18^	93.80 (1.3)	94.20 (1.2)	94.50 (1.1)	94.80 (0.9)
CJLBP^21^	88.80 (1.6)	89.20 (1.3)	89.50 (1.2)	89.88 (1.1)
LTRP^22^	87.31 (2.5)	87.66 (2.2)	87.90 (2.1)	88.20 (1.9)
LETRIST^22^	90.80 (1.6)	91.01 (1.7)	91.34 (1.6)	91.50 (1.5)

**Table 3:** Comparison of the overall percent accuracy along with the standard deviation for various descriptors, classifiers, and kernels combination with ${\mathcal {V}} = 100$, and $M = 50$.

Classifier	Kernel	inceptionv3+CT	AlexNet+CT	ResNet50+CT	Googlenet+CT
SVM	Linear	96.0% (1.2)	94.8% (1.6)	95.4% (1.1)	95.2% (1.0)
	Quadratic	95.6% (0.9)	96.2% (1.9)	95.6% (0.8)	94.8% (0.8)
	Cubic	95.4% (1.0)	96.1% (1.1)	95.2% (0.9)	94.4% (0.9)
	Fine Gaussian	58.4% (7.9)	57.8% (8.2)	58.6% (6.9)	57.4% (5.6)
	Coarse Gaussian	96.0% (0.8)	95.8% (0.9)	95.8% (1.1)	94.4% (0.8)
	Medium Gaussian	93.6% (2.3)	94.2% (2.6)	93.2% (2.0)	94.4% (1.9)
KNN	Fine	92.2% (2.4)	94.0% (2.7)	90.2% (2.3)	92.6% (2.1)
	Medium	94.6% (2.6)	94.2% (3.1)	94.4% (2.1)	93.6% (2.2)
	Coarse	91.6% (3.7)	95.0% (2.5)	93.0% (3.2)	91.8% (3.1)
	Cosine	95.8% (2.2)	94.8% (2.4)	94.4% (1.9)	93.8% (2.0)
	Cubic	92.8% (1.9)	92.4% (2.8)	91.8% (1.8)	90.2% (1.6)
	Weighted	95.2% (2.1)	94.4% (1.7)	94.2% (1.9)	94.4% (1.5)
Tree	Fine	90.8% (3.6)	88.8% (4.2)	90.4% (3.2)	90.4% (3.1)
	Medium	90.8% (2.8)	88.8% (3.3)	90.4% (2.5)	90.4% (2.2)
	Coarse	85.1% (2.6)	83.7% (2.9)	85.7% (2.5)	86.5% (2.3)
Ensemble	Bagged trees	95.6% (1.4)	96.1% (2.4)	95.6% (1.3)	94.8% (1.1)
	Boosted trees	67.3% (9.6)	66.1% (9.9)	75.7% (8.7)	83.7% (7.5)
	Subspace discriminant	96.0% (1.1)	96.2% (1.2)	95.8% (0.9)	94.6% (0.8)
	Subspace KNN	95.6% (2.2)	95.8% (1.1)	95.4% (1.0)	94.8% (1.3)
	RUSBoosted trees	74.9% (6.8)	64.9% (7.7)	75.1% (5.8)	82.7% (4.9)

Here CT denotes ColorTexture descriptor.

In Table 1, the classification performance of different SVM kernels is compared for different handcrafted features extracted models. For a fixed value of ${\mathcal {V}} = 10$ and $M=100$, different kernels of the SVM are analyzed against various descriptors. For all the SVM kernels, the ColorTexture descriptor outperformed the other three descriptors. Moreover, among the SVM kernels, the performance of the Quadratic kernel exceeded the rest of the kernels. Although the accuracy of the Linear kernel is much closer to the Quadratic kernel, while Cubic kernel provides considerably lower accuracy. For the Quadratic kernel (from right to left in Table 1), the sequence of accuracy improvement is as follows: 87.31% for LTRP, 88.80% for CJLBP, 90.83% for LETRIST, and 93.82% for ColorTexture. In terms of the standard deviation values for the SVM Quadratic kernel, moving from left to right in the table, the values are 2.5, 1.6, 1.6, and 1.3 for LTRP, CJLBP, LETRIST, and ColorTexture, respectively. Hence, ColorTexture outperformed LTRP, CJLBP, and LETRIST with an accuracy difference of 6.51%, 5.02%, and 2.99%, respectively. Similarly, the ColorTexture descriptor exhibits a higher accuracy of 93.80% with a lower standard deviation (STD) of 1.3 when using the Quadratic kernel. This is followed by the Coarse Gaussian, Linear, Cubic, Medium Gaussian, and Fine Gaussian kernels, which achieve classification accuracies of 92.80% (STD: 1.7), 92.60% (STD: 2.4), 91.00% (STD: 2.7), 92.40% (STD: 1.9), and 85.50% (STD: 3.8), respectively. It is noteworthy that the Quadratic kernel outperforms the Coarse Gaussian, Linear, Cubic, Medium Gaussian, and Fine Gaussian kernels by margins of 1.02%, 1.22%, 2.82%, 3.42%, and 8.27%, respectively.

In Table 2, the provided handcrafted descriptors are evaluated using the SVM classifier with a Quadratic kernel. The results are obtained for varying values of the cluster size ${\mathcal {V}}$ while maintaining a fixed segment size of $M = 50$. In general, the accuracy of the descriptors demonstrates an upward trend as ${\mathcal {V}}$ increases. Additionally, the ColorTexture descriptor exhibits superior performance compared to the other descriptors across all values of ${\mathcal {V}}$. For the case where ${\mathcal {V}} = 10$, the accuracy values for ColorTexture, LETRIST, CJLBP, and LTRP increase from 93.80% (STD: 1.3), 90.80% (STD: 1.6), 88.80% (STD: 1.6), and 87.31% (STD: 2.5) to 94.80% (STD: 0.9), 91.50% (STD: 1.5), 89.88% (STD: 1.1), and 88.20% (STD: 1.9), respectively, when the cluster size ${\mathcal {V}}$ is set to 100. Thus, for ${\mathcal {V}} = 100$, the ColorTexture provides higher percent accuracy than LETRIST, CJLBP, and LTRP with an accuracy difference of 3.3%, 4.92%, and 6.6% respectively. Furthermore, it is observed that the ColorTexture provides higher accuracy for the higher value of ${\mathcal {V}}$ and the best result is obtained with 1% accuracy improvement by increasing the value of ${\mathcal {V}}$ from 10 to 100.

**Table 4:** Classification performance of various standalone and proposed hybrid descriptors with ${\mathcal {V}} = 100$, $M = 50$, and SVM-Quadratic classifier.

Descriptor	Percent accuracy	F1 score	STD
ColorTexture^18^	94.80	94.93	0.67
Inception-v3^23^	87.80	87.99	1.16
ColorTexture + Inception-v3	95.60	95.82	0.90
AlexNet^19^	90.00	90.24	3.13
ColorTexture + AlexNet	96.20	96.47	1.90
ResNet 50^24^	86.50	86.65	0.93
ColorTexture + ResNet 50	95.60	95.88	0.80
GoogleNet^25^	88.80	88.92	0.94
ColorTexture + GoogleNet	94.80	94.97	0.80

Similarly, in Fig. 5, the effect of different segment sizes of the input image *M* on the percent accuracy performance of the feature extraction models is presented. As shown in Fig. 5, for most of the feature extraction models, the accuracy is higher with a patch size of M=100 compared to $M=50$ and $M=150$. It’s worth mentioning that our approach’s accuracy is influenced not only by features of photometric invariance but also by the number of utilized input features. In the conducted experiments, each image maintains dimensions of 512 × 512 pixels. Notably, when the parameter *M* is set to 50, a greater number of patches with smaller patch sizes can be extracted compared to scenarios when *M* is set to a higher value. Increasing the patch size reduces the total number of overlapped patches collected from the image, thereby reducing the accuracy of the proposed model. The reason behind this fact is that a decrease in *M* results in generating a larger quantity of patches per image. However, the increased number of patches might concurrently diminish the differences between any two patches and subsequently lead to a reduction in overall accuracy. On the other hand, a very high value of *M* leads to a smaller number of extracted patches per image, which in turn leads to a smaller number of input features and hence results in accuracy degradation. The descriptors are analyzed over the SVM classifier with Quadratic kernel, and vocabulary ${\mathcal {V}}$ remains 100. The value of *M* varies from 50 to 100. For all sets of *M*, the ColorTexture model performed better than the other feature extraction models, and the descriptors show their best for $M = 100$. However, in the case of ColorTexture, minor changes occur in classification accuracy as *M* is increased from 50 to 100, and then a decrease is observed as *M* is further increased from 100 to 150. The ColorTexture provides a larger set of features when *M* = 50 compared to *M* = 100, 150, with a minor difference in accuracy. Therefore, the patch size *M* = 50 is selected for further parametric analysis. Similarly, for $M = 50$, the ColorTexture, LETRIST, CJLBP, and LTRP give an accuracy of 94.80%, 91.50%, 89.88%, and 88.20%, respectively. Table 1 displays that the ColorTexture with a quadratic SVM kernel offers lower STD compared to other methods. Table 2 demonstrates that for ${\mathcal {V}}$ = 100, the ColorTexture descriptor achieves an accuracy of 94.80% (STD: 0.9), surpassing the LETRIST (STD: 1.5), CJLBP (STD: 1.1), and LTRP (STD: 1.9) descriptors by 3.3%, 4.92%, and 6.6%, respectively. Lastly, Table 3 concludes that the optimal combination is the ColorTexture model with $M = 50$. Therefore, the most effective combination for the provided dataset is the ColorTexture feature extraction model paired with the SVM classifier and Quadratic kernel. Furthermore, the highest accuracy is attained with ${\mathcal {V}} = 100$ vocabularies and a segment size of $M = 50$. Figure 5 clearly indicates that the ColorTexture descriptor outperforms the LETRIST, CJLBP, and LTRP descriptors.

The classification performance in terms of overall percent accuracy of various classifiers and its kernel in combination with various descriptors are presented in Table 3. Furthermore, we also present the standard deviation for k-fold validation with two iterations in each fold. It is observed from Table 3 that the SVM with Quadratic kernel in combination with the coexisted AlexNet and ColorTexture provides superior performance with an overall accuracy of 96.2%.

Considering the same parameters, we analyzed the given dataset over DNN models such as Inception-v3, AlexNet, ResNet 50, and GoogleNet. The percent accuracy performance of the standalone DNN models along with the hybrid models is listed in Table 4. It is clear from the result in Table 4 that considering hybrid models by using the handcrafted ColorTexture descriptor along with the other listed DNN provides better performance. Similarly, out of standalone DNN models such as ResNet-50, Inception-v3, GoogleNet, and AlexNet, higher performance accuracy is provided by AlexNet with an accuracy difference of 1.2%, 2.2%, and 3.5% with ResNet-50, Inception-v3, GoogleNet, respectively. Moreover, among the hybrid models using ColorTexture with AlexNet, i.e. ColorTexture+AlexNet provides better percent accuracy performance compared with ColorTexture+GoogleNet, ColorTexture+Inception-v3, and ColorTexture+ResNet 50.

**Figure 6:**
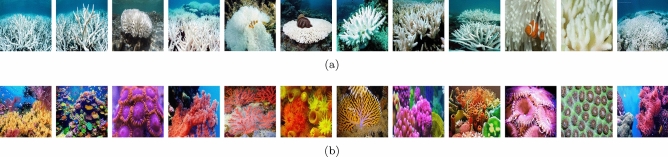
The classification performance samples of the proposed RL-BoVHF framework: (**a**) classified bleached corals images (**b**) classified unbleached corals images.

Figure 6 presents the sample images that are classified by the proposed RL-BoVHF from the input images. Figure 6a and b show the bleached and unbleached coral images, respectively, that are classified during the test of the proposed framework.

## Conclusion

In this paper, we present a highly accurate localization scheme for identifying bleached corals using the RL-BoHVF technique. Our proposed hybrid framework combines ColorTexture and AlexNet feature extraction methods while utilizing SVM with a Quadratic kernel for classification purposes. The feature dimensions of the raw data are reduced through the application of the bag-of-features approach, which in turn achieves invariance by extracting local features from image segments rather than utilizing a global representation. We have observed that, for the given dataset, the proposed RL-BoHVF model achieves high classification accuracy when the segment size (*M*) is set to 50 and the cluster size (${\mathcal {V}}$) is set to 100. The values for segment size and cluster size have been optimized through extensive experimentation. Furthermore, the classification performance demonstrates that the ColorTexture method outperforms other handcrafted descriptors, achieving an accuracy of 94.80%, with a standard deviation of 0.67. When integrating the AlexNet Deep Neural Network (DNN) into the RL-BoHVF approach, a classification accuracy of 96.20% is attained for the same dataset. However, it’s important to note that this combination results in a higher standard deviation of 1.9, indicating that the method’s robustness is somewhat compromised compared to the lower standard deviations of 0.9, 0.8, and 0.8 observed in the Inception-v3, ResNet 50, and GoogleNet models, respectively. Moving forward, our future work regarding the proposed method will focus on carefully selecting and excluding specific DNN layers that might reduce fluctuations in the final results.

## Data Availability

The datasets used and analysed in the current study are available in the Kaggle repository, Bleached and Unbleached Corals Classification.

## Abbreviations

AI: Artificial intelligence
BoF: Bag-of-features
CNN: Convolutional neural networks
DNN: Deep neural network
FCNN: Fully connected CNN
ILDP: Improved local directional pattern
KNN: K-nearest neighbor
LBPriu2: Local binary patterns with rotation invariant of uniformity less than 2
LETRIST: Locally encoded transform feature histogram
MLC: Moorea labeled corals
NOAA: National oceanic and atmospheric administration
RL- BoHVF: Robustly localization using Bag-of-hybrid visual features
SST: Sea surface temperature
SVM: Support vector machine
